# Relationship Between the Quorum Network (Sensing/Quenching) and Clinical Features of Pneumonia and Bacteraemia Caused by *A. baumannii*

**DOI:** 10.3389/fmicb.2018.03105

**Published:** 2018-12-17

**Authors:** Laura Fernandez-Garcia, Antón Ambroa, Lucia Blasco, Ines Bleriot, Maria López, Rocio Alvarez-Marin, Felipe Fernández-Cuenca, Luis Martinez-Martinez, Jordi Vila, Jesús Rodríguez-Baño, Jose Garnacho-Montero, Jose Miguel Cisneros, Alvaro Pascual, Jeronimo Pachón, German Bou, Younes Smani, Maria Tomás

**Affiliations:** ^1^Microbiology Department-Biomedical Research Institute A Coruña (INIBIC), Hospital A Coruña (CHUAC), University of A Coruña (UDC), A Coruña, Spain; ^2^Clinical Unit for Infectious Diseases, Microbiology and Preventive Medicine, Institute of Biomedicine of Seville (IBIS), University Hospital Virgen del Rocío/CSIC/University Seville, Seville, Spain; ^3^Clinical Unit for Infectious Diseases, Microbiology and Preventive Medicine, Department of Microbiology and Medicine, Biomedicine Institute of Seville, Hospital Universitario Virgen Macarena, University of Seville, Seville, Spain; ^4^Unit of Microbiology, Department of Microbiology, Maimonides Biomedical Research Institute of Cordoba, University Hospital Reina Sofía, University of Córdoba, Córdoba, Spain; ^5^Institute of Global Health of Barcelona (ISGlobal), Hospital Clínic - Universitat de Barcelona, Barcelona, Spain; ^6^Intensive Care Clinical Unit-Institute of Biomedicine of Seville (IBIS), Hospital Virgen Macarena, Seville, Spain; ^7^Department of Medicine, University of Seville, Seville, Spain

**Keywords:** quorum, sensing/quenching, pneumonia, bacteraemia, *Acinetobacter*

## Abstract

*Acinetobacter baumannii* (Ab) is one of the most important pathogens associated with nosocomial infections, especially pneumonia. Interest in the Quorum network, i.e., Quorum Sensing (QS)/Quorum Quenching (QQ), in this pathogen has grown in recent years. The Quorum network plays an important role in regulating diverse virulence factors such as surface motility and bacterial competition through the type VI secretion system (T6SS), which is associated with bacterial invasiveness. In the present study, we investigated 30 clinical strains of *A. baumannii* isolated in the “II Spanish Study of *A. baumannii* GEIH-REIPI 2000-2010” (*Genbank Umbrella Bioproject* PRJNA422585), a multicentre study describing the relationship between the Quorum network in *A. baumannii* and the development of pneumonia and associated bacteraemia. Expression of the *aidA* gene (encoding the AidA protein, QQ enzyme) was lower (*P* < 0.001) in strains of *A. baumannii* isolated from patients with bacteraemic pneumonia than in strains isolated from patients with non-bacteraemic pneumonia. Moreover, *aidA* expression in the first type of strain was not regulated in the presence of environmental stress factors such as the 3-oxo-C12-HSL molecule (substrate of AidA protein, QQ activation) or H_2_O_2_ (inhibitor of AidA protein, QS activation). However, in the *A. baumannii* strains isolated from patients with non-bacteraemic pneumonia, *aidA* gene expression was regulated by stressors such as 3-oxo-C12-HSL and H_2_O_2_. In an *in vivo Galleria mellonella* model of *A. baumannii* infection, the *A. baumannii* ATCC 17978 strain was associated with higher mortality (100% at 24 h) than the mutant, *abaI*-deficient, strain (carrying a synthetase enzyme of Acyl homoserine lactone molecules) (70% at 24 h). These data suggest that the QS (*abaR* and *abaI* genes)/QQ (*aidA* gene) network affects the development of secondary bacteraemia in pneumonia patients and also the virulence of *A. baumannii*.

## Introduction

*Acinetobacter baumannii* is a major cause of hospital-acquired infections associated with high mortality rates (Fuchs, 2016),s usually affecting patients in Intensive Care Units (ICU) (del Mar Tomas et al., 2005; Lee et al., 2017). In these patients, *A. baumannii* causes infections such as pneumonia or, to a lesser extent, serious infections of the bloodstream (around 10% of clinical isolates of *A. baumannii* cause bacteraemia) (Cisneros and Rodríguez-Baño, 2002; El Kettani et al., 2017).

The success of this bacterium as a nosocomial pathogen, has been attributed to the following factors, amongst others: (i) high genetic versatility, facilitating rapid adaptation to stressful or unfavorable situations (Gayoso et al., 2014; Trastoy et al., 2018); (ii) ability to acquire new genes horizontally by the acquisition of plasmids and phages (López et al., 2018); (iii) ability to persist for a long time on animate and inanimate surfaces (resistance to desiccation) (Gayoso et al., 2014), which is generally attributed to biofilm formation; (iv) resistance to antimicrobial agents, including broad-spectrum antibiotics such as carbapenems, colistin, and tigecycline (Fernández-Cuenca et al., 2015), as well as to disinfectants and biocides (Fernández-García et al., 2018); and (v) high virulence (colonization, invasiveness, and cytotoxicity) (Rumbo et al., 2014; Wong et al., 2017). These characteristics contribute to the fact that nosocomial outbreaks caused by *A. baumannii* are difficult to control and that therapeutic options to treat infections are scarce or non-existent (Fernández-Cuenca et al., 2013). In February, 2017, the World Health Organization (WHO) published a list of “priority pathogens.” The list includes antibiotic resistant bacteria, considered a serious threat to human health and for which new antibiotics are urgently needed, and is headed by carbapenem-resistant *A. baumannii* (Tacconelli et al., 2018).

The Quorum Sensing (QS) network is generally used by Gram-negative bacterial pathogens to regulate biological processes such as virulence, conjugation, resistance, biofilm formation (which also depends on other factors such as the lytic enzymes responsible for peptidoglycan recycling: Vijayakumar et al., 2016), motility and bacterial competition, via secretion systems (T6SS), which are associated with greater invasiveness (LaSarre and Federle, 2013; López et al., 2017a,b). Two proteins (AbaI /AbaR) identified in *A. baumannii* have been described as homologs of the LuxI/LuxR system found in *Vibrio fischeri*. This system comprises a signal or autoinducer molecule (acyl-homoserine lactone, AHL), an enzyme that synthesizes signaling molecules (AbaI) and a receptor protein activator of the QS (AbaR), which forms a complex with N-(3-hydroxydodecanoil)-L-homoserine lactone (3-OH-C12-HSL) to regulate virulence factors, biofilm formation, surface motility, and bacterial competence (T6SS) (Stacy et al., 2012). When a threshold concentration is reached, the AHL molecules present inside the cell are transported to its receptor (AbaR), putatively joining the lux-box, which is located 67 bp upstream of the ATG of AbaI, resulting in the synthesis of more AHL molecules (López et al., 2017b). The QS mechanism, on the other hand, acts naturally under environmental stress conditions such as the presence of bile salts in the gastrointestinal tract and H_2_O_2_ (ROS response) in the respiratory tract (López et al., 2017b).

A new enzyme (AidA) has recently been cloned in *E. coli* BL21 (DE3) and functionally characterized in clinical strains of *A. baumannii* capable of inhibiting their own QS (by Quorum Quenching) (López et al., 2017b). This enzyme acts by degrading signaling molecules such as N-(3-Oxo-dodecanoyl), L-homoserine lactone (3-Oxo-C12-HSL), and N-dodecanoyl-L-homoserine lactone (C12-HSL), as confirmed by observation of inhibition of motility, biofilm formation and other virulence factors associated with activation of the Quorum Sensing system (López et al., 2017b; Mayer et al., 2018). Other QQ enzymes have also recently been described in *A. baumannii* ATCC17978 (A1S_0383, A1S_2662, A1S_1876) (Mayer et al., 2018). Multiple QQ enzymes have been analyzed in diverse pathogens such as *Pseudomonas aeruginosa* (Zhang et al., 2011), *Deinococcus radiodurans, Hyphomonas neptunium, Photorhabdus luminicencens*, and *Rhizobium* spp. (Kalia et al., 2011; Krysciak et al., 2011).

Based on these findings, in the present study, we examined the relationship between the global Quorum regulatory network (QS/QQ) mediated by the *abaR* (QS) and *aidA* (QQ) genes and the development of pneumonia and bacteraemia in clinical strains of *A. baumannii* isolated in the “II Spanish Study of *A. baumannii* GEIH-REIPI 2000-2010,” a multicentre study involving 45 Spanish hospitals and 246 patients. In addition, we used an *in vivo* infection model consisting of larvae of the wax moth *Galleria mellonella* to examine the relationship between the global QS/QQ and the development of mortality by a mutant *abaI* (QS)-deficient strain of *A. baumannii* (*A. baumannii* ATCC17978Δ*abaI*) relative to that of the wild-type *A. baumannii* ATCC17978 strain.

## Materials and Methods

### Bacteria and Samples

To carry out this study, we analyzed 30 clinical strains of *A. baumannii* from the 465 strains isolated in the “II Spanish Study of *A. baumannii* GEIH-REIPI 2000-2010” multicentre study (Genbank Umbrella Bioproject PRJNA422585). The multicentre study included 45 hospitals in Spain, in which new cases of colonization or infection by *A. baumannii* were analyzed between February and March 2010 (Villar et al., 2014). The 30 *A. baumannii* strains were all isolated from respiratory samples from patients with nosocomial pneumonia (*n* = 13: 6 with and 7 without bacteraemia) or *A. baumannii* colonization of the lower respiratory tract (*n* = 17) (Sánchez-Encinales et al., 2017). Molecular typing was performed by Multilocus Sequence Typing (MLST) (Mosqueda et al., 2014). In addition, we used a killing assay with the *Galleria mellonella* infection model and an *A. baumannii* ATCC17978Δ*abaI* mutant strain (identified by Castañeda-Tamez et al., 2018).

The main clinical study variables included demographics, underlying diseases, mechanical ventilation, tracheostomy, colonization of lower respiratory airways, bacteraemic pneumonia (Pn-B), non-bacteraemic pneumonia (Pn-NB) (Horan et al., 2008) and any cause of death during hospitalization.

To design the primers and probes of the QS genes and QQ enzymes, we analyzed the presence of QS genes (*abaR* and *abaI*) and the QQ enzyme (*aidA*) in *A. baumannii* ATCC 17978 (Genbank genome accession numbers CP000521.1 [CP018664.1]) and in 1000 *A. baumannii* genomes by consulting the “Integrated Microbial Genomes and Microbiomes” web page (https://img.jgi.doe.gov) and using nucleotide BLAST. The gene sequences used in the search were selected from the *Acinetobacter baumannii* ATCC 17978 genome. A threshold of 1e-50 was used as the limit for analysis of the nucleotide sequence, where the e-value was defined as the probability of random alignments with the same score. We also calculated the percentage presence of these genes in the genomes (Figure S1).

### RNA Extraction

#### RNA Extraction to Analyze the Quorum Regulatory Network (QS/QQ)

All clinical strains of *A. baumannii* were cultured on solid Luria-Bertani (LB) plates and incubated at 37° C for 24 h. One colony was removed and inoculated in liquid LB medium and incubated overnight at 37° C under stirring at 180 rpm. The inoculum was diluted (1:100) and allowed to grow until an optical density (OD_600_ nm) of 0.4–0.6 (corresponding to the logarithmic growth phase) was reached. The RNA was then extracted using the High Pure RNA Isolation kit (Roche, Germany) and the extract was treated with Dnase (Roche, Germany). The extracted RNA was subsequently quantified in a NanoDrop ND-1000 spectrophotometer (NanoDrop Technologies), and the concentration was adjusted to 50 ng/μl in order to yield efficiencies of 90-110% (Rumbo et al., 2013). All extractions were carried out in duplicate.

#### RNA Extraction to Analyze the Quorum Regulatory Network (QS/QQ) Under Stress Conditions (3-Oxo-C12-HSL and H_2_O_2_)

The 13 strains of *A. baumannii*, isolated from patients with pneumonia, were cultured on solid Luria-Bertani (LB) plates and incubated at 37°C for 24 h. One colony was then removed, inoculated in liquid LB medium and incubated overnight at 37°C under stirring at 180 rpm. The preinoculum was diluted (1:100) and allowed to grow until an optical density (OD600 nm) of 0.3 was reached. Aliquots of 10 μM of 3-Oxo-C12-HSL (QS-inactivating molecule by expression of the AidA protein) (Stacy et al., 2012; López et al., 2017b) and (10 μl) H_2_O_2_ were then added for 5 min (QS-activator by ROS response) (López et al., 2018). All controls were prepared by adding the same volumes of DMSO (dimethyl sulfoxide), 3-Oxo-C12-HSL and of sample, but with no H_2_O_2_. After incubation of the samples for 4 and 5 h in the presence of 3-Oxo-C12-HSL, to study the regulatory QS/QQ genes (*abaR* and *aidA*), as well as 5 min under H_2_O_2_ in static at 37°C, RNA was extracted using the High Pure RNA Isolation kit (Roche, Germany) and treated with Dnase. The extracted RNA was subsequently quantified as described above (Rumbo et al., 2013).

### RT-qPCR

The studies were carried out with a Lightcycler 480 RNA MasterHydrolysis Probe (Roche, Germany), under the following conditions: reverse transcription at 63°C for 3 min, denaturation at 95°C for 30 s, followed by 45 cycles of 15 s at 95°C and 45 s at 60°C and, finally, cooling at 40°C for 30 s. The UPL primers and probes from conserved DNA regions identified by PCR (Universal Probe Library-Roche, Germany) used in the analysis are shown in Table 1.

**Table 1:** Primers and Probes used in this study.

		**Sequence (5′-3′)**	**Probe**	**Reference**
**QUORUM SENSING**				
*abaR*	Forw	TGGCAAGAAGATTTATTATCAGCA	119/TTGGTGGT	This study
	Rev	TGCGGTAGATTTAACGATCTCA		
	Forw	AGAGGCGTTACGTTGGACTG	155/GAAGGCAA	This study
	Rev	CCAAGAATCTGAGCTATTGC		
**QUORUM QUENCHING**				
*aidA*	Forw	GGGAACTTCTTTCGGTGGAG	145/CAGCGACC	López et al., 2017b
	Rev	AACAGCAGCAAGTCGATTATCA		
	Forw	CCTAACCTTGCATTAGGGCTATTA	53/TGGCAGAG	López et al., 2017b
	Rev	CGGTAAACCACAGGTCGGTA		
**HOUSEKEEPING**				
*rpoB*	Forw	CGTGTATCTGCGCTTGG	131/CTGGTGGT	Rumbo et al., 2014
	Rev	CGTACTTCGAAGCCTGCAC		

All of the experiments were carried out in a final volume of 20 μl per well (18 μl of master mix and 2 μl of RNA). Each experiment was carried out in duplicate with two RNA extracts (50 ng/μl). For each strain, the expression of all genes, primers, and probes was normalized relative to the reference or housekeeping gene, *rpoB*, for RT-qPCR studies of Quorum sensing Primer sequences (5′-3′) with Taqman probes (Rumbo et al., 2013; López et al., 2017b). Analysis of the controls without reverse transcriptase confirmed the absence of DNA contamination.

### *Galleria mellonella* Infection Model

The *Galleria mellonella* model was an adapted version of that developed by Peleg et al. (2009), Yang et al. (2015). The procedure was as follows: twelve *G. mellonella* larvae, acquired from TruLarvTM (Biosystems Technology, Exeter, Devon, UK), were each injected with 10 μl of a suspension of *A. baumannii* ATCC17978, or its isogenic deficient mutant *A. baumannii* ATCC17978Δ*abaI*, diluted in sterile phosphate buffer saline (PBS) and containing 8 × 10^4^ CFU (± 0. 5 log). The injection was performed with a Hamilton syringe (volume 100 μl) (Hamilton, Shanghai, China). In addition, a control group of twelve larvae were injected with 10 μl of sterile PBS. After being injected, the groups of larvae were placed in Petri dishes and incubated in darkness at 37°C. The number of dead larvae was recorded twice a day (morning and afternoon) for 6 days. The larvae were considered dead when they showed no movement in response to touch (Peleg et al., 2009).

### Statistical Analysis

The gene expression studies were carried out in duplicate, and the data obtained were analyzed by Student's *t*-test, implemented with GraphPad Prism v.6 software (GraphPad Software Inc. San Diego, CA). The graphs were constructed using the GraphPad program, and the results were represented as means and their respective standard deviations.

The mortality curves corresponding to the *in vivo Galleria mellonella* infection model were constructed using GraphPad Prism v.6 and the data were analyzed using the Log-rank test (Mantel-Cox). In both cases, *p*-values < 0.05 were considered statistically significant, and the data were expressed as mean values.

The statistical analyses were applied to the following categorical variables: age, sex, immunosuppressive treatment, surgery, ICU stay, mechanical ventilation, tracheostomy, severe sepsis, septic shock, and expression of the Quorum genes in *A. baumannii* clinical strains (Bone et al., 1992). In addition, the severity of co-morbidities was assessed using the Charlson score (Charlson et al., 1987) and the McCabe score (McCabe and Jackson, 1962). Chi-square and Fisher tests were used in the univariate analysis of categorical variables. Continuous variables were analyzed using two-sample *t*-test or Mann Whitney, as appropriate. A logistic regression analysis was performed to identify factors independently associated with pneumonia and bacteraemia. Differences were considered significant at *p* < 0.05. All statistical analyses were performed using SPSS v.16.0 (SPSS Inc., Chicago, IL).

## Results

### Study of the Gene Expression of the *abaR* and *aidA* Genes of the Quorum Network (QS/QQ)

**Figure 1:**
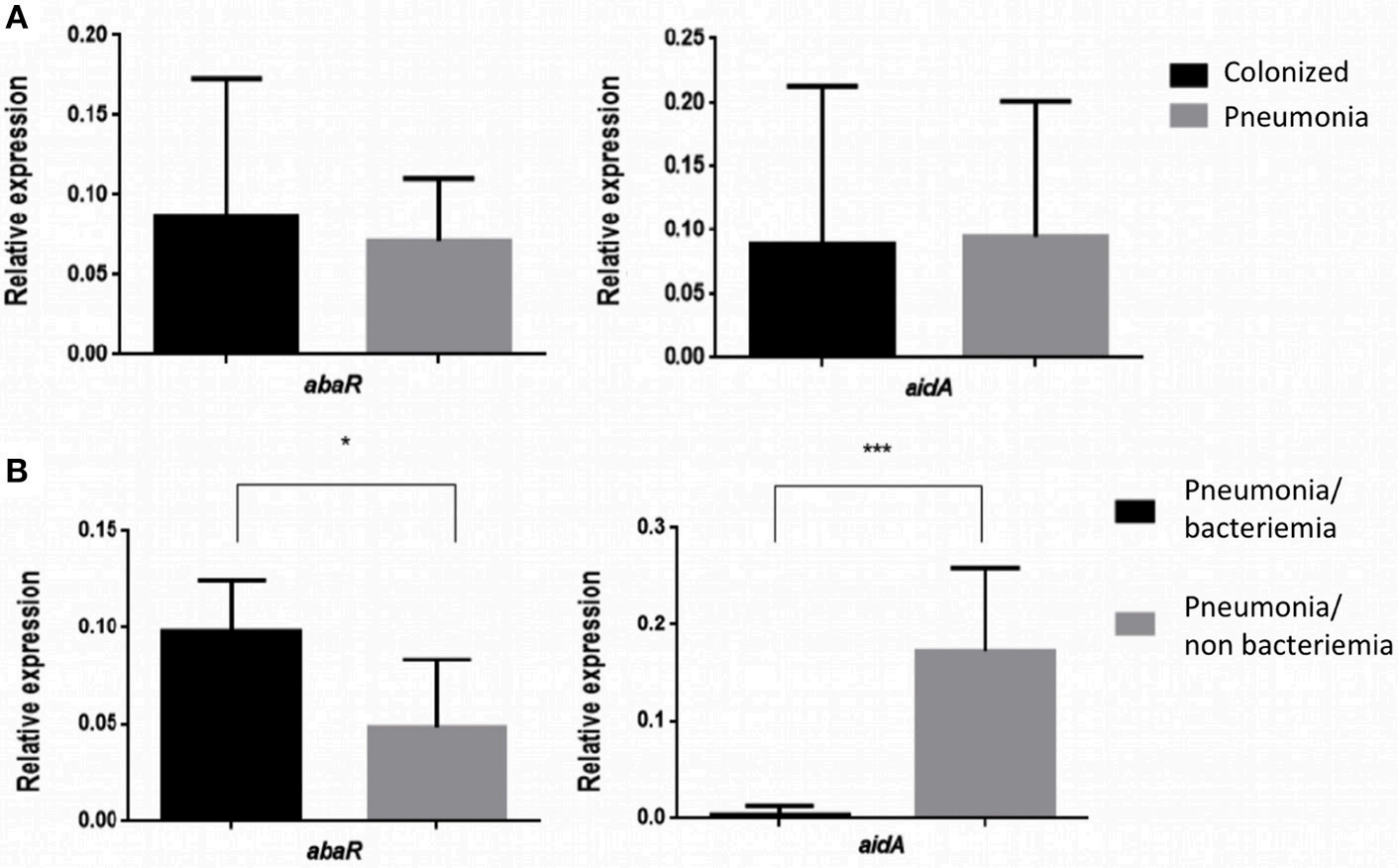
**(A)** Relative Expression of the *abaR* and *aidA* genes in strains of *A. baumannii* from patients colonized with *A. baumannii* and patients with pneumonia caused by *A. baumannii*. No significant differences (*p* > 0.05) were detected in either case. **(B)** Relative expression of the *abaR* and *aidA* genes in isolates of *A. baumannii* from patients with bacteraemic and non-bacteraemic pneumonia. ^*^p-value < 0.05 and ^***^p-value < 0.001.

The Relative Expression (RE) of the *abaR* and *aidA* genes of the Quorum network (QS/QQ) was quantified by RT-qPCR analysis of the 17 isolates of *A. baumannii* from colonized patients and of the 13 isolates of *A. baumannii* from patients with pneumonia (Figure 1A). The mean values (of two biological replicates) are presented in Tables 2, 3. These values were first used to determine any significant differences between the two types of strains in terms of gene expression in the Quorum network.

**Table 2:** Results of RT-qPCR analysis of the Relative Expression (RE) of the *abaR* and *aidA* genes (Quorum network genes) in the *A. baumannii* isolates from colonized patients.

**Strain (MLST^a^)**	***abaR* (RE)**	***aidA* (RE)**
**STRAINS OF** ***A. baumannii*** **ISOLATED FROM COLONIZED PATIENTS**		
Ab 22_GEIH-2010 (ST-52)	0.043	0.042
Ab 38_GEIH-2010 (ST-2)	0.078	0.146
Ab 59_GEIH-2010 (ST-269)	0.081	0.003
Ab 64_GEIH-2010 (ST-2)	0.076	0.213
Ab 77_GEIH-2010 (ST-261)	0.018	0.112
Ab 112_GEIH-2010 (ST-263)	0.112	0.038
Ab 141_GEIH-2010 (ST-264)	**0.001**	0.010
Ab 177_GEIH-2010 (ST-2)	**0.001**	0.067
Ab 205_GEIH-2010 (ST-2)	0.131	0.126
Ab 288_GEIH-2010 (ST-263)	0.067	0.199
Ab 290_GEIH-2010 (ST-264)	0.141	0.006
Ab 294_GEIH-2010 (ST-2)	0.123	0.481
Ab 326_GEIH-2010 (ST-2)	0.123	0.015
Ab 354_GEIH-2010 (ST79)	0.052	0.020
Ab 364_GEIH-2010 (ST-79)	**0.001**	0.081
Ab 399_GEIH-2010 (ST-79)	0.061	0.050
Ab 456_GEIH-2010 (ST-269)	0.368	**0.001**

*The results are expressed as the mean value of the two biological replicates*.

a *MLST (Mutilocus Sequence Typing by Pasteur database, https://pubmlst.org/) (Villar et al., 2014). In bold, RE ≤ 0.001 not detected by RT-PCR*.

**Table 3:** Results of RT-qPCR analysis of the Relative Expression (RE) of the *abaR* and *aidA* genes (Quorum network genes) in the *A. baumannii* isolates from patients with bacteraemic pneumonia (Pn-B) or non-bacteraemic pneumonia (Pn-NB).

***A. baumannii*** **strains from Pn-NB patients**			***A. baumannii*** **strains from Pn-B patients**		

**Strain (MLST^a^)**	***abaR*** **(RE)**	***aidA*** **(RE)**	**Strain (MLST^a^)**	***abaR*** **(RE)**	***aidA*** **(RE)**
Ab 8_GEIH-2010 (ST-2)	0.035	0.108	Ab 148_GEIH-2010 (ST-2)	0.094	0.022
	0.592^*^	1.545^*^		1.032^*^	1.204^*^
	0.940^**^	0.873^**^		1.407^**^	0.598^**^
Ab 73_GEIH-2010 (ST-2)	0.036	0.361	Ab 215_GEIH-2010 (ST-2)	0.085	**0.001**
	0.564^*^	1.695^*^		0.883^*^	0.001^*^
	0.763^**^	0.450^**^		1.376^**^	0.001^**^
Ab 125_GEIH-2010 (ST-257)	0.047	0.156	Ab 232_GEIH-2010 (ST-2)	0.139	**0.001**
	0.431^*^	1.582^*^		0.692^*^	0.001^*^
	1.366^**^	1.076^**^		0.845^**^	0.001^**^
Ab 157_GEIH-2010 (ST-2)	**0.001**	0.172	Ab 275_GEIH-2010 (ST-181)	0.110	**0.001**
	0.329^*^	2.308^*^		0.638^*^	0.001^*^
	1.272^**^	0.685^**^		1.430^**^	0.001^**^
Ab 240_GEIH-2010 (ST-2)	0.078	0.150	Ab 371_GEIH-2010 (ST-79)	0.059	**0.001**
	0.561^*^	0.858^*^		0.503^*^	0.001^*^
	0.553^**^	0.703^**^		1.233^**^	0.001^**^
Ab 268_GEIH-2010 (ST-181)	0.034	0.139	Ab 461_GEIH-2010 (ST-2)	0.099	**0.001**
	0.683^*^	1.530^*^		1.221^*^	0.001^*^
	0.845^**^	0.717^**^		2.713^**^	0.001^**^
Ab 276_GEIH-2010 (ST-181)	0.108	0.126			
	0.553^*^	1.007^*^			
	0.586^**^	0.722^**^			

*The results are expressed as the mean values of the two biological replicates*.

a *MLST (Mutilocus Sequence Typing by Pasteur database, https://pubmlst.org/) (Villar et al., 2014). In bold, RE ≤ 0.001, not detected by RT-PCR*.

* * Results of RT-qPCR analysis of the Relative Expression (RE) of the abaR/aidA genes (Quorum network genes) in the presence of 3-Oxo-C12-HSL in strains of A. baumannii from patients with bacteraemic pneumonia (Pn-B) or non-bacteraemic pneumonia (Pn-NB)*.

** * Results of RT-qPCR analysis of the Relative Expression (RE) of the abaR/aidA genes (Quorum network genes) in the presence of H_2_O_2_ in strains of A. baumannii from patients with bacteraemic pneumonia (Pn-B) or non-bacteraemic pneumonia (Pn-NB)*.

The results did not reveal any significant differences in the RE of the Quorum network genes (*abaR, aidA*) between clinical strains of *A. baumannii* isolated from colonized patients and strains of *A. baumannii* isolated from patients with pneumonia (0.086/0.094 vs. 0.071/0.095, *p* > 0.05).

We then proceeded to study the RE of the *abaR* and *aidA* genes in strains of *A. baumannii* from patients with pneumonia, differentiating the strains isolated from patients with bacteraemia (Pn-B) from those isolated from patients without bacteraemia (Pn-NB). The resulting graphs are shown below (Figure 1B). The findings reveal significant differences in the expression of the *abaR* and *aidA* genes between clinical strains of *A. baumannii* from patients with bacteraemic pneumonia (Pn-B) and those with non-bacteraemic pneumonia (Pn-NB). We observed that *abaR* gene was overexpressed in *A. baumannii* isolates from Pn-B patients relative to Pn-NB patients (0.047 vs. 0.097, *p* < 0.05). By contrast, the *aidA* gene was overexpressed in *A. baumannii* clinical strains in Pn-NB patients relative to Pn-B patients (0.173 vs. 0.0045, *p* < 0.001) (Figure 1B). Only one strain, Ab 148_GEIH-2010 (ST-2), isolated from Pn-B patients, showed an *aidA* gene profile different from the other isolates of this group, although the RE of this gene was lower (0.022) than that of isolates from Pn-NB patients.

### Study of *abaR*/*aidA* Genes (QS/QQ) Under Stress Conditions (3-Oxo-C12-HSL and H_2_O_2_)

**Figure 2:**
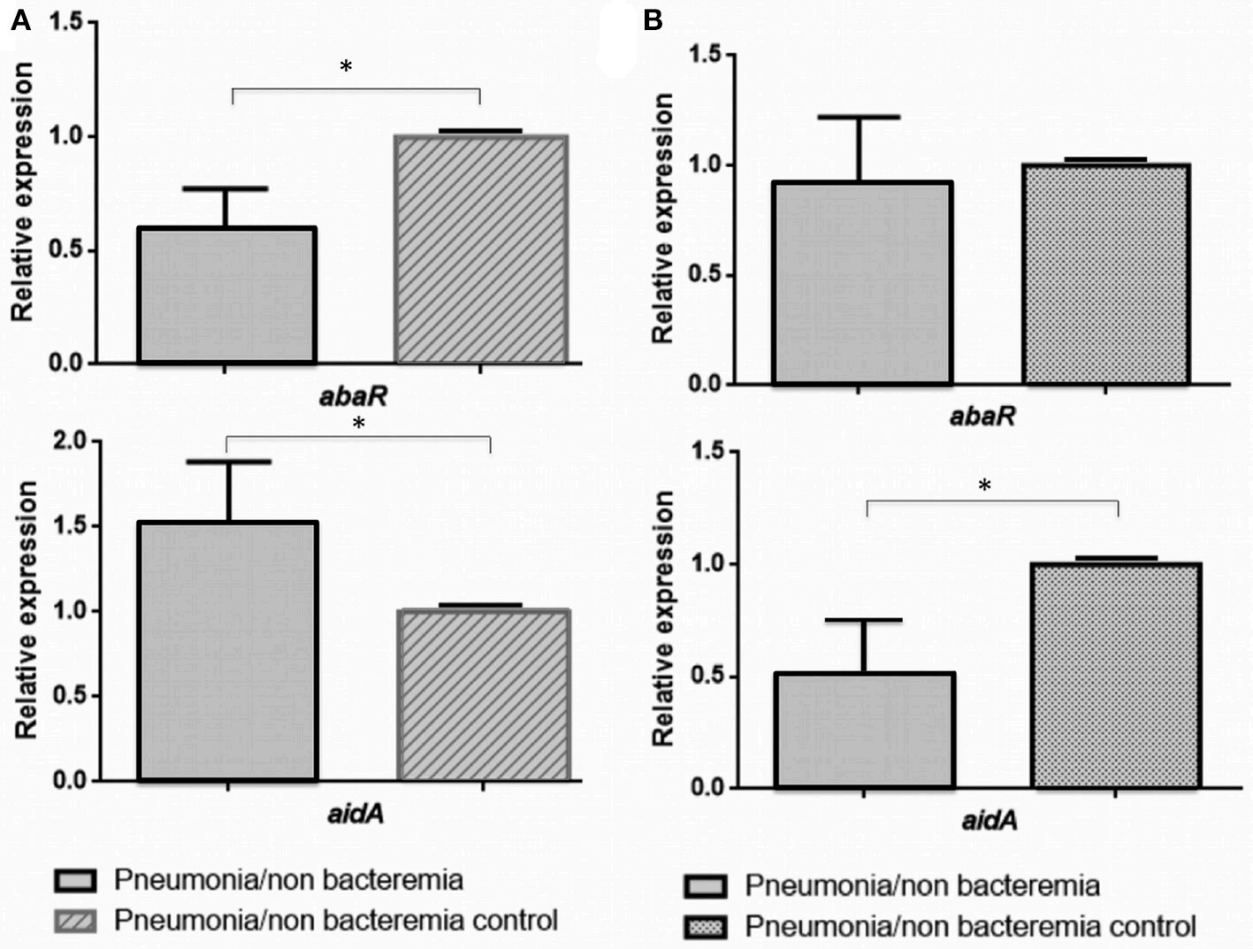
Relative expression of the *abaR* and *aidA* genes under 3-oxo-C12-HSL **(A)** and H_2_O_2_ **(B)** in isolates of *A. baumannii* from patients with non-bacteraemic pneumonia (Pn-NB). ^*^p-value < 0.05.

**Figure 3:**
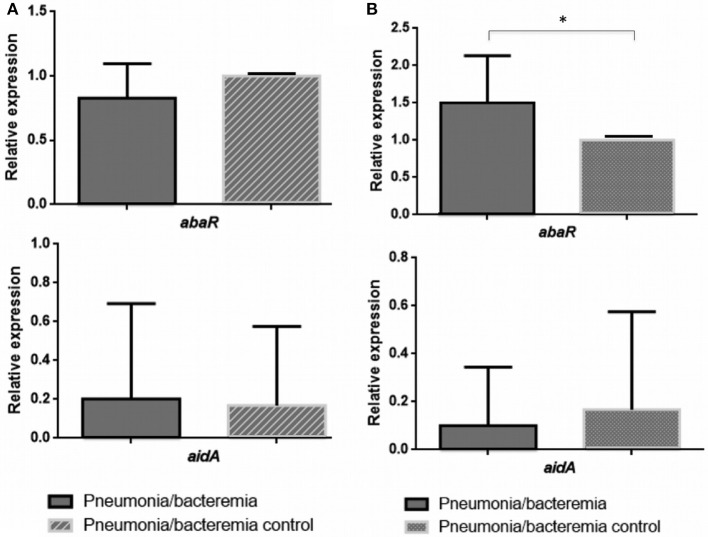
Relative expression of the *abaR* and *aidA* genes under 3-oxo-C12-HSL **(A)** and H_2_O_2_ **(B)** in isolates of *A. baumannii* from patients with bacteraemic pneumonia (Pn-B). ^*^p-value < 0.05.

The values of the RE of the *abaR* and *aidA* genes (Quorum network) in the presence of 3-Oxo-C12-HSL (Inhibition of the QS) and H_2_O_2_ (Activation of the QS), obtained by RT-qPCR of the 13 isolates of *A. baumannii* from patients with pneumonia (differentiated from Pn-NB) are shown in Table 3, expressed as the mean value of the two biological replicates. These values were then analyzed to determine any significant differences in the RE of the *abaR/aidA* (QS/QQ) genes between the different clinical isolates (Figures 2, 3).

In the clinical strains of *A. baumannii* isolated from Pn-NB (Figure 2), we observed regulation of expression of the *aidA* gene in the presence of 3-Oxo-C12-HSL (overexpression, RE ≥ 1.5) (Figure 2A) and of H_2_O_2_ [underexpression, RE ≤ 0.5 (Figure 2B)]. Expression of the *abaR* gene decreased significantly in the presence of the 3-Oxo-C12-HSL molecule (RE ≤ 0.5, Figure 2A).

In the clinical strains *A. baumannii* isolated from Pn-B (Figure 3), expression of the *aidA* gene was not regulated in the presence of 3-Oxo-C12-HSL or H_2_O_2_. However, the *abaR* gene was overexpressed in the presence of H_2_O_2_ (RE ≥ 1.5, Figure 3B).

These results indicate that the isolates of *A. baumannii* from Pn-NB may harbor a functional AidA protein (QQ enzyme), in contrast to the isolates of *A. baumannii* from Pn-B, which did not have this functional protein. Therefore, in the *A. baumannii* strains isolated from Pn-B, overexpression of the *abaR* gene (activation of the QS) in the presence of H_2_O_2_ (ROS response) would enable the development of the virulence factors favoring invasiveness, such as type VI secretion system (T6SS) and motility.

### Quorum Network (QS/QQ) Genes and Clinical Variables

Analysis of the risk factors associated with the development of pneumonia vs. colonization by clinical strains of *A. baumannii* revealed only one statistically significant variable, i.e., diabetes mellitus (Table 4).

**Table 4:** Univariate analysis of risk factors associated with development of pneumonia relative to colonization by clinical strains of *A. baumannii*.

**Variable**	**Colonized patients (*n* = 17)**	**Patients with pneumonia (*n* = 13)**	***P*-value**
Age, Med ± SEM	55.06 ± 5.12	59.31 ± 5.90	0.590
Female sex	5 (29.41)	6 (46.15)	0.287
Charlson score, Med ± SEM	2.12 ± 0.67	2.46 ± 0.69	0.729
Comorbity condition, no. (%)			
McCabe score, ultimately or rapidly	8 (47.06)	5 (38.46)	0.840
Cancer	1 (5.88)	2 (15.38)	0.397
**Diabetes**	**3 (17.65)**	**7 (53.85)**	**0.045**
Cirrhosis	0 (0)	0 (0)	NA
AIDS	0 (0)	1 (7.69)	0.433
Chronic lung disease	2 (11.67)	4 (30.78)	0.204
CRF	0 (0)	1 (7.69)	0.433
Immunosuppression	1 (5.88)	2 (15.38)	0.397
Surgery, No. (%)	5 (29.41)	4 (30.78)	0.623
ICU stay, No. (%)	15 (88.23)	11 (84.61)	0.591
Tracheostomy	4 (23.53)	2 (15.38)	0.469
Mechanical ventilation	10 (58.82)	7 (53.85)	0.538
Death	3 (17.65)	3 (23.08)	0.531
*abaR*	0.09 ± 0.02	0.07 ± 0.04	0.547
*aidA*	0.09 ± 0.03	0.09 ± 0.03	0.919

*In bold and highlighted the variables that showed a p < 0.05*.

However, analysis of the risk factors associated with the development of bacteraemia in pneumonia caused by *A. baumannii* revealed underexpression of the *aidA* gene as the only statistically significant variable (*p* < 0.05) (Table 5).

**Table 5:** Univariate analysis of risk factors associated with the development of bacteraemia in pneumonia caused by *A. baumannii* relative to the non-bacteraemic pneumonia control.

**Variable**	**Pn-NB patients (*N* = 7)**	**Pn-B patients (*N* = 6)**	***P*-value**
Age, Med ± SEM	58.43 ± 10.13	60.33 ± 6.08	0.880
Female sex	4 (57.14)	2 (33.33)	0.383
Charlson score, Med ± SEM	2.00 ± 0.79	3.20 ± 1.24	0.497
Comorbity condition, no. (%)			
McCabe score, ultimately or rapidly	3 (42.86)	2 (33.33)	0.380
Cancer	1 (14.28)	1 (20)	0.731
Diabetes	2 (28.57)	5 (83.33)	0.078
Cirrhosis	0 (0)	0 (0)	NA
AIDS	0 (0)	1 (20)	0.462
Chronic lung disease	3 (42.86)	1 (20)	0.343
CRF	1 (14.28)	0 (0)	0.538
Immunosuppression	2 (28.57)	0 (0)	0.269
Surgery, No. (%)	3 (42.86)	1 (20)	0.343
ICU stay, No. (%)	5 (71.43)	6 (100)	0.269
Tracheostomy	0 (0)	2 (33.33)	0.192
Mechanical ventilation	3 (42.86)	4 (66.67)	0.383
Severe sepsis and septic shock, No. (%)	2 (28.57)	3 (50)	0.565
Death	3 (42.86)	0 (0)	0.122
*abaR*	0.78 ± 0.015	0.06 ± 0.02	0.522
***aidA***	**0.15 ± 0.04**	**0.03 ± 0.03**	**0.045**

*In bold and highlighted the variables that showed a p < 0.05*.

### Mortality in the *in vivo Galleria mellonella* Model

**Figure 4:**
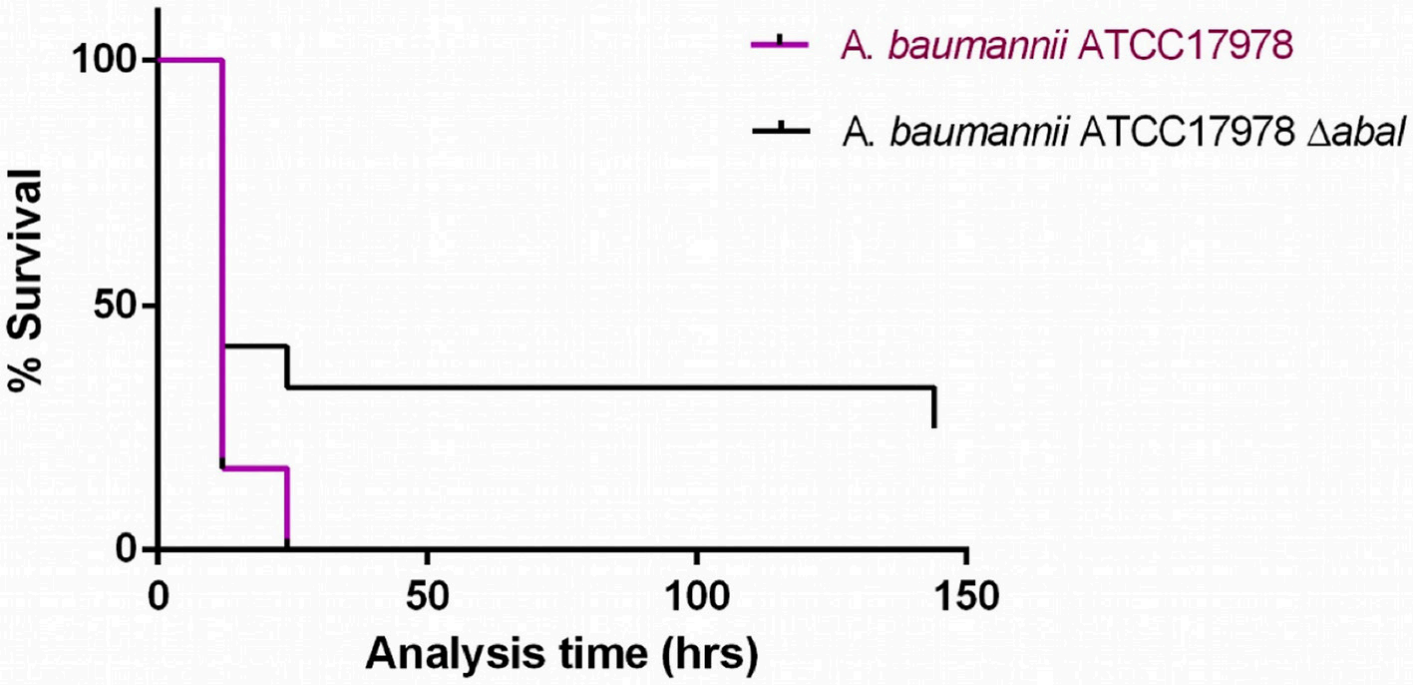
Survival curves for *G. mellonella* larvae injected with *A. baumannii* ATCC17978 reference strain and its isogenic derivative *A. baumannii* ATCC17978 Δ*abaI*. Data from a single representative assay. For simplicity, the control group is not included in this figure.

Injection of *G. mellonella* larvae with *A. baumannii* ATCC17978 at a concentration of 8 x 10^4^ CFU/larva (± 0.5 log) caused 100% mortality after 24 h, whereas injection of the larvae with the same concentration of *A. baumannii* ATCC17978Δ*abaI* resulted in 70% mortality after 24 h (Figure 4; *p* < 0.05, Mantel-Cox analysis).

## Discussion

In this study, we analyzed the expression of Quorum network (QS/QQ) genes that differed between genomes of clinical isolates of *A. baumannii, abaR* and *abaI* (QS system) and *aidA* (QQ mechanism) in relation to clinical features of pneumonia and bacteraemia. Although other QQ enzymes have been described in *A. baumannii* ATCC 17978 (Mayer et al., 2018), these were not analyzed in the present study due to the lack of any differences between *A. baumannii* genomes.

In clinical strains of *A. baumannii* isolated from patients with bacteraemic pneumonia (Pn-B), the *abaR* gene was overexpressed (*p* < 0.05). The AbaR protein was the receptor activator of the Quorum Sensing system (QS), and the *aidA* gene was not expressed. Moreover, we observed regulation of *aidA* gene expression in clinical strains of pneumonia-causing *A. baumannii* (non-bacteraemic pneumonia, Pn-NB) by the 3-Oxo-C12-HSL molecule (which is an AidA enzyme substrate in QQ activity) and H_2_O_2_ (an activator of the QS system). However, there was no difference in the expression of Quorum network genes between colonized and pneumonia patients, as previously described (Stones and Krachler, 2016).

On the other hand, clinical analysis of the risk factors associated with pneumonia caused by *A. baumannii* revealed diabetes mellitus as only statistically significant risk factor (Kim et al., 2014). In relation to bacteraemia in *A. baumannii* pneumonia (*P* < 0.05), underexpression of the *aidA* gene was also the only statistically significant variable (*P* < 0.05).

In several pathogens, such as *Yersinia pseudotuberculosis, Proteus mirabilis*, and *Vibrio cholerae*, the QS system is the main regulatory mechanism of bacterial competence via T6SS, which is involved in the invasiveness and motility that favor the development of bacteraemia (Zhang et al., 2011; Debnath et al., 2018; Jaskólska et al., 2018; Trastoy et al., 2018). Moreover, in 86% of ICU patients, gastrointestinal tract colonization by a clinical strain of *A. baumannii* led to development of bacteraemia caused by genetically similar strains (Thom et al., 2010). This implies that clinical isolates of *A. baumannii* most capable of surviving under stress conditions (such as the presence of bile salts in the gastrointestinal tract or H_2_O_2_ in the respiratory tract) (Zheng et al., 2018) may have a higher invasive capacity due to virulence factors, such as the type VI secretion system (T6SS), previously activated under stressful conditions. Motility is also a crucial virulence factor, allowing penetration of the bacteria into the host's body and subsequent colonization (Gellatly and Hancock, 2013). Previous studies have demonstrated the existence of a relationship between motility and the origin of the isolates. Indeed, blood isolates of *A. baumannii* have been found to be more mobile than sputum isolates (Vijayakumar et al., 2016). Interestingly, 67% of the clinical isolates of *A. baumannii* were non-mobile and all of them had the AidA protein and were of respiratory origin (López et al., 2017a,b). In addition, the *aidA* gene was not located in the genome of the only mobile strain (clone ST79/PFGE-HUI-1) isolated from blood and which was the origin of a bacteraemic outbreak (López et al., 2017a,b).

Finally, multiple studies carried out with the *abaI* mutant of the M2 strain of *Acinetobacter nosocomialis* have analyzed the role of the *abaI* gene (responsible for the synthesis of quorum sensing synthesizing molecules) in various virulence factors such as biofilm formation and motility. In both cases, *abaI* deficiency led to a decrease in biofilm production and motility (Niu et al., 2008; Bhargava et al., 2012). The mutant lacking *abaI* is believed to be less virulent than the wild strain. This result was confirmed in our study in which injection of *G. mellonella* larvae with the reference *A. baumannii* ATCC17978 strain caused higher mortality than injection with the mutant *A. baumannii* ATCC17978Δ*abaI*. Regarding the mortality of the reference strain (*A. baumannii* ATCC17978), similar effects have been observed in other studies, in which injection of *G. mellonella* larvae with the reference strain *A. baumannii* ATCC17978 resulted in rapid death. Mortality was significantly dependent on the number of cells injected. More than 75% of the larvae died in the first 48 h of injection with at least 3.7 × 10^5^ CFU / larva, while very few of the larvae died after being injected with a concentration equal to or lower than 3.7 × 10^4^ CFU/larva (*p* < 0.01) (Clemmer et al., 2011). The results regarding the mutant *A. baumannii* ATCC17978Δ*abaI* are consistent with those obtained in a study of *Pseudomonas aeruginosa* (Steindler et al., 2009) in which a mutant Δ*rhLI* Δ*lasI* (QS systems homologous to *abaI*) was obtained, demonstrating that inactivation of both QS systems leads to a significant reduction in pathogenicity (*p* < 0.01) when virulence factors are not activated, such as the type VI secretion system (T6SS) and motility (Jaskólska et al., 2018).

In conclusion, our findings suggest that the QS (*abaR* and *abaI* genes)/QQ (*aidA* gene) network plays a role in the development of bacteraemia in patients with pneumonia caused by *A. baumannii*. This is the first study reporting a relationship between reduced expression of this bacterial QQ enzyme gene (AidA protein) and bacteraemia. Further studies of this relationship in the same and other bacterial QQ enzymes would be of great interest.

## Author Contributions

LF-G, AA, LB, IB, ML and RA-M developed the experiments. FF-C, LM-M, JV, JR-B, JG-M, JMC, AP, JP, GB, and YS wrote the manuscript and provided the strains. MT led the experiments and manuscript redaction.

### Conflict of Interest Statement

The authors declare that the research was conducted in the absence of any commercial or financial relationships that could be construed as a potential conflict of interest.
