# Supplementary material for: Relationship Between the Quorum Network (Sensing/Quenching) and Clinical Features of Pneumonia and Bacteraemia Caused by A. baumannii
Source: Front Microbiol. 2018 Dec 17;9:3105. doi: 10.3389/fmicb.2018.03105 (PMC6304438; doi:10.3389/fmicb.2018.03105)
Supplement: Supplementary file 1 [file Image_1.pdf]

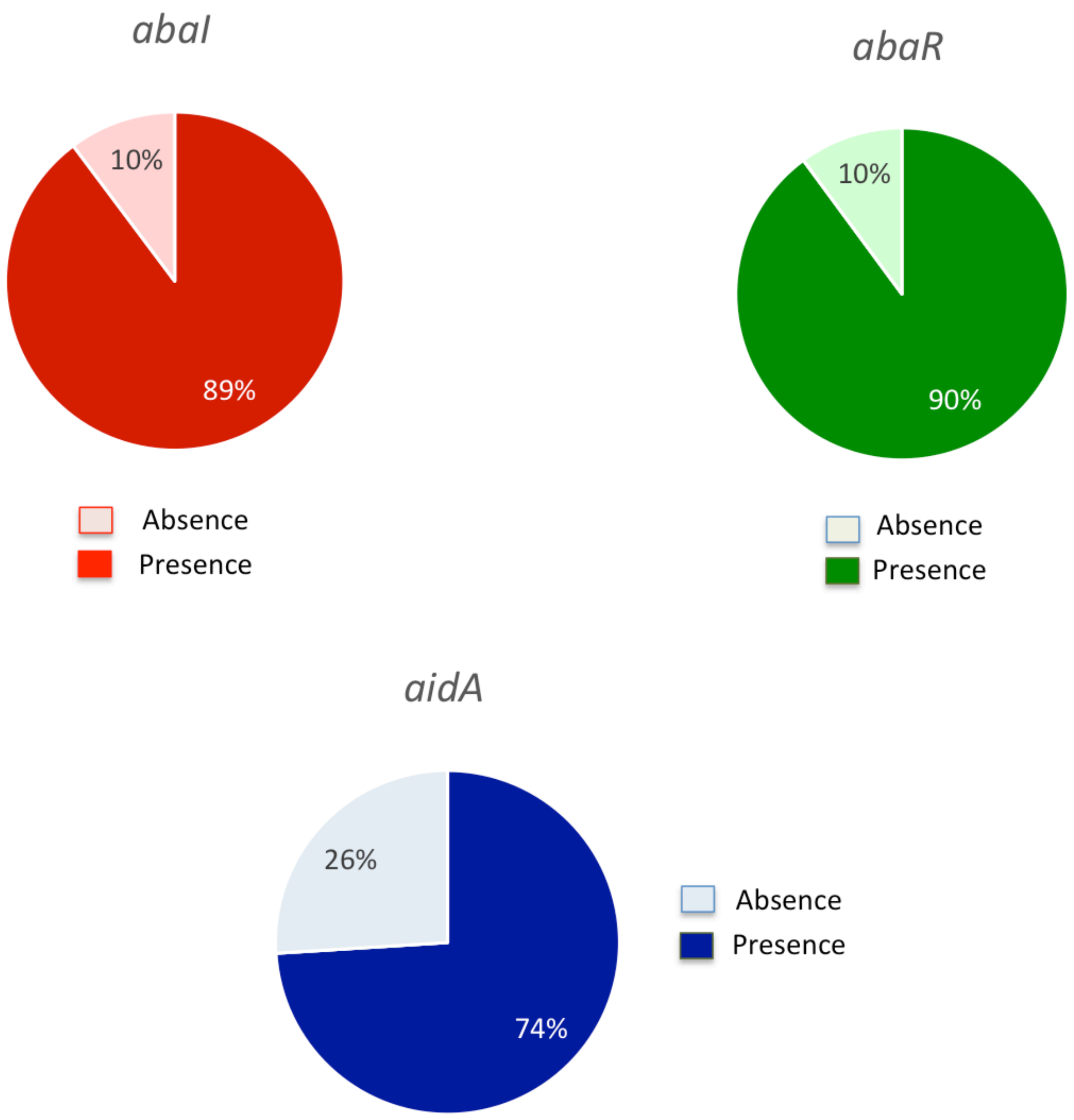

Figure 1S. Analysis of genes involved in the Quorum Sensing system (*abaR* and *abaI*) and Quorum Quenching system (*aidA*) in *A. baumannii* ATCC 17978 (Genbank genome access numbers CP000521.1 [CP018664.1]) and in 1000 *A. baumannii* genomes.
